# Study on a Two-Dimensional Scanning Micro-Mirror and Its Application in a MOEMS Target Detector

**DOI:** 10.3390/s100706848

**Published:** 2010-07-16

**Authors:** Chi Zhang, Zheng You, Hu Huang, Guanhua Li

**Affiliations:** State Key Laboratory of Precision Measurement Technology and Instruments, Department of Precision Instruments and Mechanology, Tsinghua University, Beijing 100084, China; E-Mails: yz-dpi@mail.tsinghua.edu.cn (Z.Y.); huanghu08@mails.tsinghua.edu.cn (H.H.); lgh@mails.tsinghua.edu.cn (G.L.)

**Keywords:** MOEMS, scanning mirror, piezoresistor, target detection, laser ranging

## Abstract

A two-dimensional (2D) scanning micro-mirror for target detection and measurement has been developed. This new micro-mirror is used in a MOEMS target detector to replace the conventional scanning detector. The micro-mirror is fabricated by MEMS process and actuated by a piezoelectric actuator. To achieve large deflection angles, the micro-mirror is excited in the resonance modes. It has two degrees of freedom and changes the direction of the emitted laser beam for a regional 2D scanning. For the deflection angles measurement, piezoresistors are integrated in the micro-mirror and the deflection angles of each direction can be detected independently and precisely. Based on the scanning micro-mirror and the phase-shift ranging technology, a MOEMS target detector has been developed in a size of 90 mm × 35 mm × 50 mm. The experiment shows that the target can be detected in the scanning field and the relative range and orientation can be measured by the MOEMS target detector. For the target distance up to 3 m with a field of view about 20° × 20°, the measurement resolution is about 10.2 cm in range, 0.15° in the horizontal direction and 0.22° in the vertical direction for orientation.

## 1. Introduction

With the rapid development of the micro-optical-electro-mechanical systems (MOEMS) technology, the micromation of the payloads in micro-satellites has become a general trend [1]. For optical scanning and target detection, the laser scanning technique is an active way to detect objects and measure both range and orientation [2]. The two-dimensional scanning micro-mirror has great advantages over the conventional scanning mechanisms, such as low power consumption, small volume and high frequency. It has a broad range of space applications for target detection in micro-satellites. At present, most of scanning micro-mirrors have been driven by electrostatic, electromagnetic or piezoelectric force. They have a spring structure and are operated on a resonant mode for high-speed scan operation with large deflection [3–7]. For target detection and location, the scanning micro-mirror requires the measurement of deflection angles with high sensitivities, which most of the current researches have not involved. A LIDAR (LIght Detection And Ranging) system with a magnetic mirror and a micro shutter array is adopted for planetary explorer [2], but the mechanism is complicated and the scanning field is narrow. A laser range finder coupled with two silicon micro-mirrors are used in the compact robotics perception system [8], but adopting a PSD sensor for position detection decreases the integration of the system. A MEMS electromagnetic optical scanner for a laser scanning microscope integrated sensing coil for deflection angle measurement [9], but the scanner is one-dimensional and the induced electromotive force is low with limited sensitivity.

With the aim of improving these deficiencies, a two-dimensional scanning micro-mirror with piezoresistor sensors for measurement of deflection angles is developed in this paper. It has a simple structure and a small volume, with a large scanning field and high sensitivities. Based on the scanning micro-mirror and the phase-shift ranging technology, a MOEMS target detector has also been developed. In the last section, the performance of the prototype and experimental results will be described in detail.

## 2. Two-Dimensional Scanning Micro-Mirror

### 2.1. Structure

**Figure 1.**
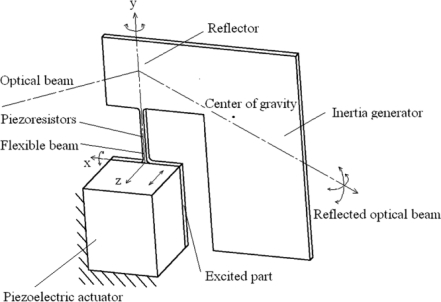
Structure of the two-dimensional scanning micro-mirror.

A two-dimensional scanning micro-mirror with a piezoelectric actuator and piezoresistors was designed as shown in Figure 1. The micro-mirror structure consists of a reflector, an inertia generator, a flexible beam and an excited part, which is 8 mm × 8 mm × 0.2 mm in size. The reflector and inertia generator are formed together, and linked with the excited part by the flexible beam. The excited part is connected to the piezoelectric actuator. The piezoresistors are integrated on the surface of the flexible beam for deflection angles measurement [10].

**Figure 2.**
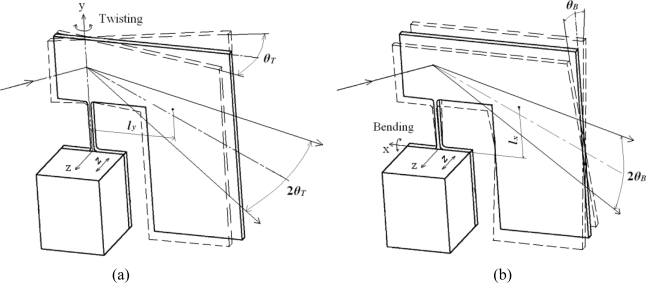
Two resonance vibration modes. **(a)** twisting around the y-axis, **(b)** bending around the x-axis.

The piezoelectric actuator deforms along the z-axis by pulsant driving voltage and the excited part vibrates in the z-axis. As the center of gravity of the reflector and inertia generator is away from each rotational axis (x and y), the micro-mirror has two resonance vibration modes: twisting around the y-axis and bending around the x-axis, as shown in Figure 2. The two-dimensional micro-mirror is thus equivalent to a two dimensional vibration system with two different resonant frequencies. Actuating the micro-mirror at each resonant frequency can make the mirror vibrate with large deflection angles *θ_T_* and *θ_B_* around the y-axis and x-axis, respectively. When a resultant voltage including two different resonant frequencies is imposed to the piezoelectric actuator, both vibration modes are excited and the micro-mirror is capable of scanning a light beam two-dimensionally with large scanning angles with a single driving source.

### 2.2. Piezoresistors

Deflection angle sensing is based on the piezoresistive effect, which has the advantages of favorable dynamic characteristics and high sensitivities. The surface stresses generated on the flexible beam when the micro-mirror is twisting or bending and piezoresistors are laid on the flexible beam for the deflection angles measurement of two directions. The change of the resistance in piezoresistor is related to the stresses and the piezoresistive coefficients in longitudinal, transverse and tangential directions. The piezoresistive effect in plane can be described as follows [11]:

(1) $$\frac{\Delta R}{R}=\pi_{l}\sigma_{l}+\pi_{t}\sigma_{t}+\pi_{\tau }\sigma_{\tau },$$

where *σ_l_* is the longitudinal stress, *σ_t_* is the transverse stress and *σ_τ_* is the tangential stress. *π_l_* is the longitudinal coefficient, *π_t_* is the transverse coefficient and *π_τ_* is the tangential coefficient.

**Figure 3.**
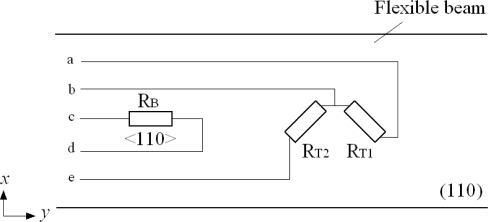
Directions and crystal orientations of piezoresistors.

**Figure 4.**
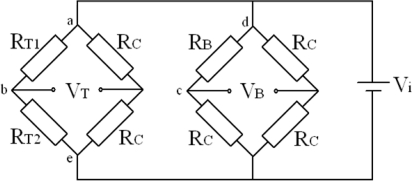
Piezoresistors connection of two Wheatstone bridges.

In order to realize the decoupling measurement for two deflection angles and obtain the large piezoresistive coefficients for the high measurement sensitivities, an n-type silicon substrate in (110) wafer is selected for the flexible beam. Two p-type silicon piezoresistors *R_T_*_1_ and *R_T_*_2_ are oriented along ±45 degrees off y-axis and a p-type silicon piezoresistor *R_B_* is oriented along y-axis in <110> crystal orientation. The directions and crystal orientations of piezoresistors and the connection of two Wheatstone bridges are shown in Figure 3 and Figure 4. With the design of piezoresistors, the deflection angles of micro-mirror in the two directions can be measured by the two Wheatstone bridges and indicated by the output voltages *V_T_* and *V_B_*, respectively [12].

### 2.3. Fabrication

**Figure 5.**
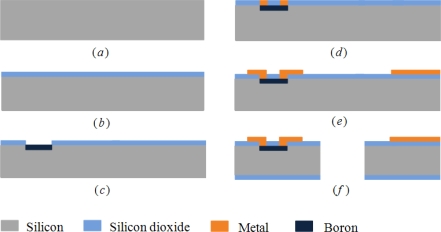
Fabrication process flows. **(a)** n-type Si substrate, **(b)** deposit SiO_2_, **(c)** diffuse p-type piezoresistors, **(d)** metallization, **(e)** sputter and lift-off, **(f)** ICP dry etching.

**Figure 6.**
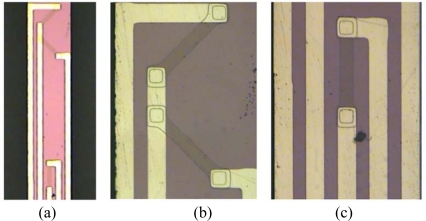
Micrographs of flexible beam and piezoresistors. **(a)** the flexible beam, **(b)** piezoresistors *R_T_*_1_ and *R_T_*_2_, **(c)** piezoresistor *R_B_*.

The fabrication process flows are shown in Figure 5. The two-dimensional scanning micro-mirror is fabricated using a bulk silicon process, starting with an n-type silicon substrate of 300 μm thickness (Figure 5(*a*) and (*b*)). Boron doping produces p-type piezoresistors on the surface (Figure 5(*c*)). In order to obtain the desired resistivity r_0_ = 1.1 × 10^−2^ Ω cm, the dimensions of the piezoresistors are set to 100 μm × 10 μm with 0.5 μm depth and the boron ion implantation density is 8.0 × 10^18^ ions/cm^2^ at the temperature of 1,100 °C. After depositing a layer of silicon dioxide again on the top of the Boron-doped region and metallization (Figure 5(d)), the sputter and lift-off process was adopted and golden thin film lines with a width of 10 μm are connected and laid on the flexible beam (Figure 5(*e*)). The micrographs of the flexible beam and piezoresistors are shown in Figure 6. Finally, the micro-mirror structure is released by inductive coupled plasma (ICP) dry etching (Figure 5(*f*)). In addition, the piezoelectric actuator is fabricated by precision machining and connected to the excited part by the epoxy resins with high strength and adhesion.

**Figure 7.**
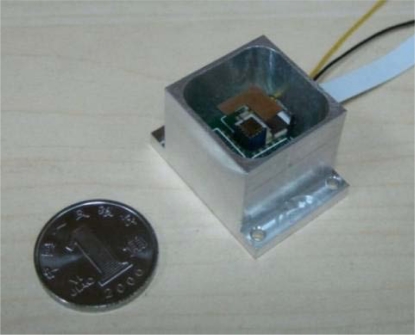
Package of two-dimensional scanning micro-mirror.

The micro-mirror is packaged in a stainless steel case with the size of 28 mm × 20 mm × 18 mm as shown in Figure 7. The top of the package is open for the reflector and it can be closed with a translucent optical glass. The actuation and detection signal are applied and recovered through access points on the package side.

### 2.4. Characteristics

**Figure 8.**
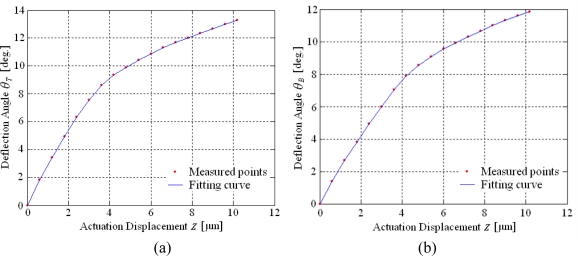
Deflection angles characteristics in two directions. **(a)** deflection angle *θ*_T_ in twisting direction, **(b)** deflection angle *θ*_B_ in bending direction.

The two resonance frequencies of the two-dimensional scanning micro-mirror are 216.8 Hz and 464.8 Hz, respectively, which are measured by the frequency sweeping method and a laser interferometer measurement system. The relationships between each deflection angle and the actuation displacement in the resonance modes are shown in Figure 8.

**Figure 9.**
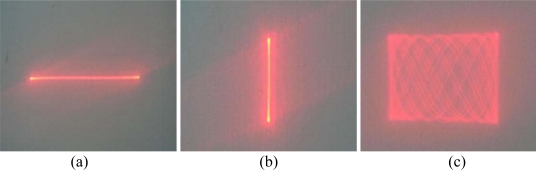
Scan patterns of two-dimensional scanning micro-mirror. **(a)** Twisting by y-axis, **(b)** Bending by x-axis, **(c)** Two-dimensional scan.

The experimental results indicate that the deflection angles become larger as the actuation displacement increases. By an actuation displacement of about 10 μm, the deflection angles twisting along the y-axis and bending on the x-axis are 13.3° × 11.8°. By reflecting the optical beam, the scanning field of the two-dimensional scanning micro-mirror is above 26° × 23°. The scan patterns of the twisting by y-axis, bending by x-axis and two-dimensional scan are shown in Figure 9.

**Figure 10.**
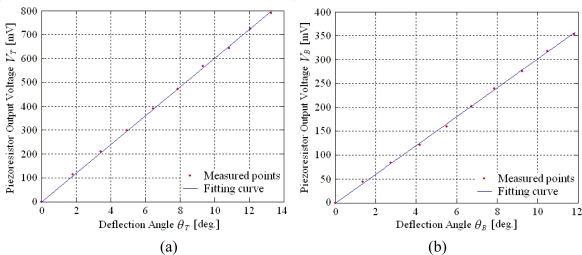
Piezoresistor output characteristics in two directions. **(a)** deflection angle measurement in twisting direction, **(b)** deflection angle measurement in bending direction.

The relationships between the corresponding piezoresistor output voltage and each deflection angle in the resonance modes are shown in Figure 10. There are linear relationships between each output voltage and each deflection angle. The deflection angles measurement sensitivities for two directions are 59 mV/deg and 30 mV/deg, respectively.

## 3. MOEMS Target Detector

### 3.1. Structure and Beam Path

**Figure 11.**
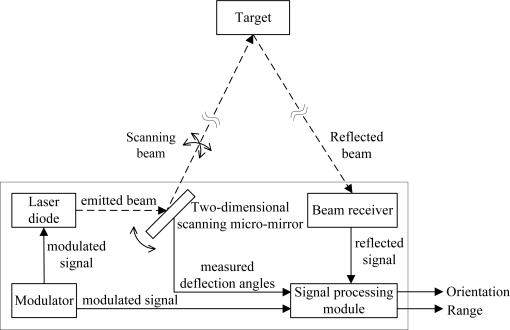
Structure of MOEMS target detector.

Based on the two-dimensional scanning micro-mirror, the MOEMS target detector has the ability of target detection and location measurement, which is mainly composed of a laser diode, a modulator, a two-dimensional scanning micro-mirror, a beam receiver and a signal processing module as shown in Figure 11. In the MOEMS target detector, the CW laser beam is collimated and emitted from the laser diode by the modulator. The emitted beam is reflected by the two-dimensional scanning micro-mirror for a regional 2D scanning. The beam reflected from the target is received by the beam receiver and converted to the reflected signal. With the contrast between the modulated signal and the reflected signal, the relative range of the target is calculated by the phase-shift ranging method [13]. With the capture time of the reflected signal and the real-time measured deflection angles of micro-mirror, the relative orientation of the target is calculated accordingly. Therefore, the target can be located by the MOEMS target detector.

**Figure 12.**
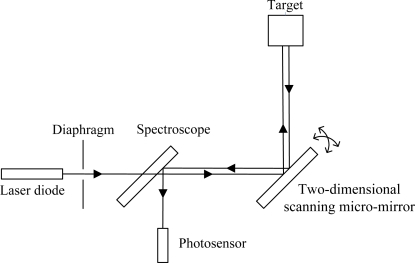
Beam path of MOEMS target detector.

In order to realize the coaxial beam path in the MOEMS target detector, the optical configuration of the system is shown in Figure 12. The emitted beam from the laser diode passes through the diaphragm and the spectroscope. The through part is reflected by the micro-mirror and scanned two-dimensionally. The return beam reflected back from the target passes through the same beam path to the spectroscope and the reflected part is detected by the photosensor.

### 3.2. Target Location Method

When the micro-mirror is scanning two-dimensionally, the deflection angles in the two directions are measured by the two Wheatstone bridges with the decoupling measurement method. The real-time measurement results can describe the relationship between the deflection angles and the time [14]. When the reflected beam is received by the photosensor, the optical signal is converted into the reflected signal, and the relative orientation of the target can be calculated by Equation (2).

(2) $$\left(\psi_{B},\psi_{T})=2\;[\theta_{B}(t),\theta_{T}(t)\right]$$

where *ψ_B_* and *ψ_T_* are the two-dimensional azimuth angles of the target, *θ_B_*(*t*) and *θ_T_*(*t*) are the real-time deflection angles of the micro-mirror in two directions, and *t* is the capture time of the reflected signal.

The target ranging is based on the phase-shift laser ranging method. The phase difference between the modulated signal and the reflected signal contains target range information. By amplification, mixing, band-pass and sampling, the phase difference is acquired by the phase meter [14], and the target range *D* is represented by Equation (3).

(3) $$D=\frac{c\cdot (\phi_{B}-\phi_{A})}{4\pi f},$$

where *φ_A_* is the phase of the modulated signal, *φ_B_* is the phase of the reflected signal, *c* is the velocity of light and *f* is the modulated frequency of the modulator.

Therefore, based on the scan orientation and laser ranging method, the target is located in the three-dimensional space by the combination of the measurement results *ψ_B_*, *ψ_T_* and *D*.

## 4. Experimental Results

### 4.1. Detector Prototype

**Figure 13.**
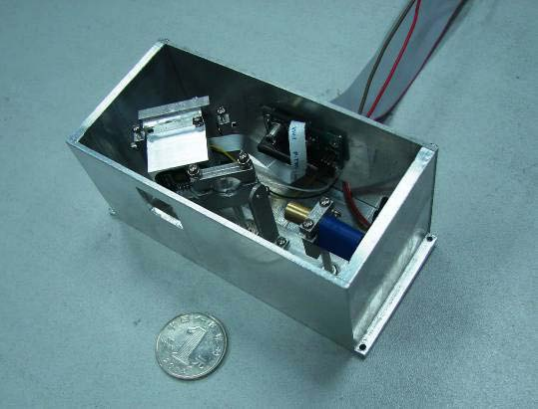
Prototype of MOEMS target detector.

Figure 13 shows the prototype of the MOEMS target detector. Modulated, emitted, scanning and received parts are integrated into a compact package, giving a size of 90 mm × 35mm × 50 mm. The two-dimensional scanning micro-mirror is located behind the front window from which the scanning beam and reflected beam are emitted and received. The photosensor is integrated in a PCB, which is located behind the spectroscope. The power and data signals are imported and exported through the back window of the MOEMS target detector.

### 4.2. Target Detection and Location

**Figure 14.**
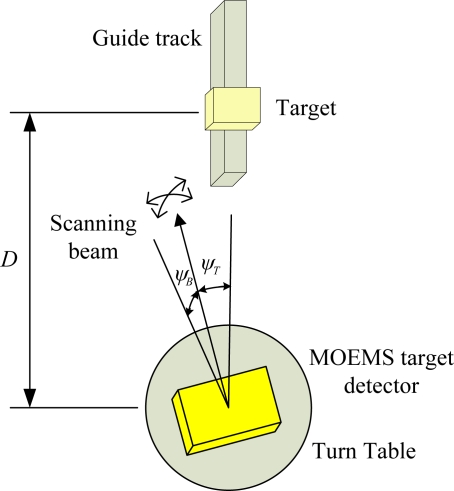
Experimental system for MOEMS target detector.

The experimental system is composed of MOEMS target detector, turn table, guide track and target, which are set on the optical vibration isolation platform as shown in Figure 14. The relative azimuth angles *ψ_B_* and *ψ_T_* of the target are varied in the scope of ±10° in the horizontal and vertical directions, by turning the turn table. The relative range *D* of the target is varied in the range of 0 to 3 m, by moving the target in the guide track.

**Figure 15.**
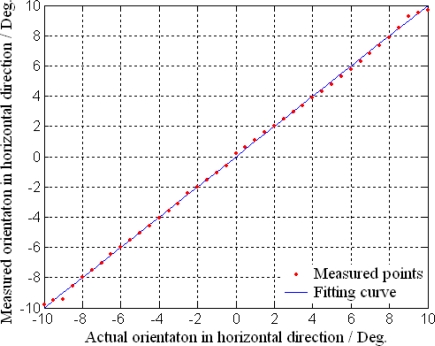
Orientation measurement in horizontal direction.

**Figure 16.**
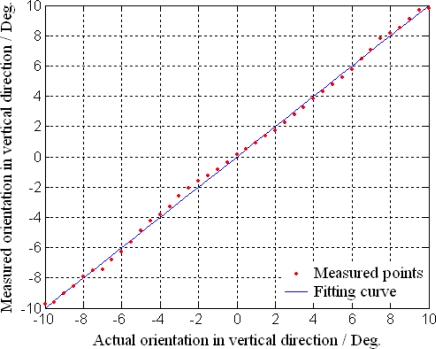
Orientation measurement in vertical direction.

The measurement results of relative orientation in the horizontal and vertical directions are shown in Figure 15 and Figure 16, respectively. The results indicate that the MOEMS target detector can receive the reflected beam from the target precisely and measure the orientation accordingly. In the scope of ±10°, the measured orientations are consistent with the actual orientations, which can verify the design and principle of the orientation measurement. The measurement precisions are 0.15° in the horizontal direction and 0.22° in the vertical direction. The orientation errors are mainly due to the measurement errors of the deflection angles for their correspondences. In the process of piezoresistors, the inaccuracy of lithography, exposure and diffusion lead to the inconsistent piezoresistors and low stabilities in the Wheatstone bridges.

**Figure 17.**
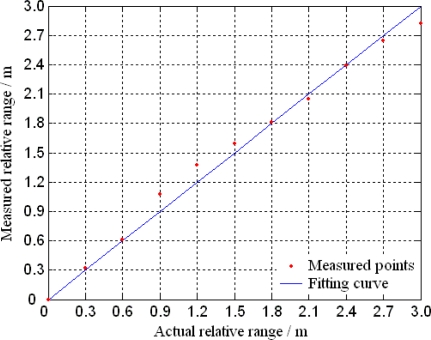
Relative range measurement.

According to Equation (3), the maximum measurement range is limited to 75 m with *f* = 2 MHz. The actual measurement range in the experiment is 3 m (14.4deg phase shift). The measurement results of the relative range are shown in Figure 17. The results indicate that the MOEMS target detector can realize the measurement of the relative range by the scanning of the micro-mirror. In the range of 0 to 3 m, the measured ranges are consistent with the actual ranges, which can verify the design and principle of the range measurement. The range error is 10.2 cm, which is mainly due to the signals conversion and sampling in the signal processing module of the detector.

Setting the target on some example locations and combining the measurement results of orientation and range, the actual location and the measured location are contrasted in Table 1. The experiment results indicate that the MOEMS target detector based on the two-dimensional scanning micro-mirror can measure the orientation and range of the target simultaneously and the target location can be exactly achieved by the scanning measurement method.

## 5. Conclusions

To replace the conventional scanning detector with optical MEMS technology, a two-dimensional scanning micro-mirror has been developed in this paper. The micro-mirror has the capabilities of regional scanning in coupled vibration modes and deflection angles measurement by the piezoresistors. The structure, piezoresistors, fabrication and characteristics of the micro-mirror are detailed. Based on the two-dimensional scanning micro-mirror and the phase-shift ranging technology, a MOEMS target detector has been developed in the size of 90 mm × 35 mm × 50 mm. The design and measurement principle are described and the experiment results show that the target can be detected in the scanning field and the relative range and orientation can be measured by the MOEMS target detector. For the target distance up to 3 m with a field of view about 20° × 20°, the measurement resolution is about 10.2 cm for range while 0.15° in the horizontal direction and 0.22° in the vertical direction for orientation. The MOEMS target detector, based on the two-dimensional scanning micro-mirror, has the great advantages of small volume, high frequency, large deflection angles and high measurement sensitivities. It is suitable for target location and has a wide foreground in the field of space detection and target identification in micro-satellites.

## Figures and Tables

**Table 1.** Target location measurements.

Target location	Horizontal orientation/ Vertical orientation/ Relative range	
	Actual location	Measured location
P1	−3° / 2° / 0.4 m	−3.11° / 1.82° / 0.43 m
P2	0° / 0° / 0.8 m	0.13° / 0.19° / 0.87 m
P3	2° / 5° / 1.2 m	2.00° / 4.84° / 1.37 m
P4	5° / 0° / 1.6 m	4.89° / 0.19° / 1.65 m
